# Can phase angle from bioelectrical impedance analysis associate with neuromuscular properties of the knee extensors?

**DOI:** 10.3389/fphys.2022.965827

**Published:** 2022-08-11

**Authors:** Kosuke Hirata, Mari Ito, Yuta Nomura, Tsukasa Yoshida, Yosuke Yamada, Ryota Akagi

**Affiliations:** ^1^ Faculty of Sport Sciences, Waseda University, Saitama, Japan; ^2^ Airweave Inc., Aichi, Japan; ^3^ Graduate School of Engineering and Science, Shibaura Institute of Technology, Saitama, Japan; ^4^ Section of Healthy Longevity Research, National Institute of Health and Nutrition, National Institutes of Biomedical Innovation, Health, and Nutrition, Tokyo, Japan; ^5^ College of Systems Engineering and Science, Shibaura Institute of Technology, Saitama, Japan

**Keywords:** bioelectrical impedance spectroscopy, quadriceps femoris, aging, sarcopenia, electromyography, twitch contractile properties, rate of torque development, dynapenia

## Abstract

Maintenance and improvement of neuromuscular functions is crucial for everyone regardless of age. An easy way to assess neuromuscular properties without muscle contraction is useful especially for those who cannot perform strenuous muscular force production, such as older adults and patients with orthopedic or cognitive disorders. Bioelectrical impedance analysis (BIA) can assess body electrical properties e.g., phase angle (PhA) which is regarded as muscle quantity/quality index. The purpose of this study was to investigate associations of PhA with neuromuscular properties of the knee extensors in 55 young (*n* = 23) and older (*n* = 32) adults. The values of PhA of the right thigh and whole-body were determined with BIA at 50 kHz. The participants performed 4-s maximal voluntary isometric contraction (MVIC) to measure peak torque (PT_MVIC_), and 1-s brief MVIC to assess rate of torque development (RTD) over the time interval of 0–200 ms. As markers of physiological mechanisms of muscle force production, twitch contractile properties (peak twitch torque, rate of twitch torque development, and time-to-peak twitch torque) of the knee extensors obtained by femoral nerve electrical stimulation, and muscle activity assessed as root mean square values of electromyographic activity (EMG-RMS) during PT_MVIC_ and RTD measurements were measured. Thigh and whole-body PhA significantly correlated with PT_MVIC_ (*r* ≥ 0.555, *p* < 0.001) and electrically evoked twitch parameters (peak twitch torque, rate of twitch torque development, and time-to-peak twitch torque; |*r*| ≥ 0.420, *p* ≤ 0.001), but not RTD (*r* ≤ 0.237, *p* ≥ 0.081) or EMG-RMSs (|*r*| ≤ 0.214, *p* ≥ 0.117). Stepwise multiple linear regression analysis revealed that thigh PhA was selected as a significant variable to predict PT_MVIC_ but not RTD. Whole-body PhA was not selected as a significant variable to predict PT_MVIC_ or RTD. In conclusion, both thigh and whole-body PhA can associate with maximal voluntary muscle strength of the knee extensors, and this association may be due to intrinsic contractile properties but not neural aspects. Regarding prediction of the knee extensor strength, thigh PhA is preferable as the predictor rather than whole-body PhA which is used as a widely acknowledged indicator of sarcopenia.

## Introduction

Bioelectrical impedance analysis (BIA) is a widely acknowledged method to estimate body composition. BIA quickly and non-invasively measures tissue electrical parameters (resistance and reactance) by sending a weak electric current through the body. Resistance is inversely related to the water and electrolyte content of tissue, and reactance is positively associated to the capacitance properties of the cell membrane (Barbosa-Silva et al., 2005). Phase angle (PhA) is a measure obtained with BIA, and calculated as [arctangent (reactance/resistance) × 180°/π]. Considering this equation and characteristics of resistance and reactance as mentioned above, larger PhA expects to larger amount of high-water content tissue (e.g., muscle cell) and better cell membrane integrity. Indeed, larger PhA is regarded as an indicator of larger muscle cell mass (Di Vincenzo et al., 2021) and better cellular membrane integrity (Schwenk et al., 2000). PhA is also known to be an index of muscle function. For instance, previous studies reported that larger PhA evaluated for whole-body associated with greater hand grip strength in adolescents and young adults (Sacco et al., 2021), and faster 5-m walking speed in older adults (Yamada et al., 2019). Although muscle function can be assessed from physical performance tests and/or muscle strength measurements using a dynamometer in case of healthy population, there are those who cannot perform physical test or forceful muscle contraction, such as infants, elderly adults, injured people, or dementia patients. For everyone especially those people, PhA seems to be a desirable measure to assess muscle function, because PhA can be quickly and non-invasively assessed by BIA without any muscle force generation.

Previous studies revealed that PhA correlated with maximal muscle strength, such as hand grip strength (Uemura et al., 2019; Yamada et al., 2019; Sacco et al., 2021), and ankle plantar flexion and dorsiflexion strength (Yoshida et al., 2018). Regarding age-associated muscle strength loss, decline in thigh muscle strength including the knee extensor strength is suggested to be greater than strength loss of the other muscle group (Frontera et al., 2000; Nogueira et al., 2013). Because the knee extensors are considered to play an important role for daily physical activity (Hasegawa et al., 2008), it is worthwhile for older adults to know whether PhA can associate with knee extension strength or not. Also, since knee extension strength is obviously important to athletic performance (such as sprint, jump, and kick), quick assessment of knee extension strength must be valuable even for young adults. However, there is no study to investigate the association of PhA with knee extension strength in healthy adults by basic single-joint test using dynamometer, while the statistically significant correlation between PhA and isometric knee extension torque was reported in the patients with knee osteoarthritis (Wada et al., 2020). Recently, Yamada et al. (2021) have suggested that locomotor function evaluated by timed-up-and-go test is more highly correlated with PhA measured from lower limb compared with PhA measured from whole-body or arm. Although whole-body PhA is used to judge sarcopenia/dynapenia (Cruz-Jentoft et al., 2019; Yamada et al., 2019), knee extension strength, which is highly susceptible to age-related muscle atrophy (Frontera et al., 2000; Nogueira et al., 2013), may be less associated with whole-body PhA. If so, whole-body PhA may not be the best way to assess or detect sarcopenia/dynapenia.

Maintaining higher maximal muscle strength is important for superior athletic performance and independence in the later life of individuals. In addition, explosive muscle strength (ability of rapid force generation) is also essential for everyone regardless of age, since explosive muscle strength associates with sprint and agility performance (Wang et al., 2016), and activities of daily living such as rising from a chair and stair walking (Aagaard et al., 2010), balance control (Ema et al., 2017), and the risk of falls (Rubenstein, 2006). Although, as mentioned earlier, several studies revealed the correlation between PhA and maximal muscle strength (Yoshida et al., 2018; Uemura et al., 2019; Yamada et al., 2019; Sacco et al., 2021), it has not been explored whether PhA can associate with explosive muscle strength. Furthermore, underlying mechanism of association between PhA and muscle strength has been unclear. Hence, investigating the association coupled with influential factors of muscle strength [e.g., twitch contractile properties, neuromuscular activity, muscle architecture, and musculotendinous stiffness (Maffiuletti et al., 2016)], may be important to comprehend why PhA relates with muscle strength. PhA is suggested to relate to cellular membrane function (Schwenk et al., 2000; Barbosa-Silva et al., 2005). In addition, a previous study (Yamada et al., 2022) reported that membrane capacitance of the leg evaluated by bioelectrical impedance spectroscopy associated with twitch torque and electromyography (EMG) amplitude of the plantar flexors. Considering with close relationship among impedance variables (e.g., PhA and membrane capacitance) (Yamada et al., 2010), PhA may reflect twitch contractile properties and neuromuscular activity which are affected at least in part by cellular membrane function.

We aimed to investigate 1) whether thigh PhA associates with maximal muscle strength, explosive muscle strength, twitch contractile properties, and neuromuscular activity in knee extensors of healthy young and older adults or not, and 2) which PhA (thigh PhA or whole-body PhA) is the best predictor of muscle strength and neuromuscular function of the knee extensors. Hypotheses of the present study were 1) larger thigh PhA would correlate with greater maximal muscle strength, explosive muscle strength, twitch contractile properties, and neuromuscular activity in the knee extensors, and 2) thigh PhA would be the preferable predictor of maximal muscle strength, explosive muscle strength, twitch contractile properties, and neuromuscular activity in the knee extensors rather than whole-body PhA.

## Materials and methods

### Participants

*A priori* power analysis was performed to compute the sample size for correlation analysis using G*Power statistical power analysis software (G*Power 3.1.9.7; Kiel University, Germany). Referring previous studies (Yoshida et al., 2018; Uemura et al., 2019; Yamada et al., 2019; Sacco et al., 2021), effect size was assumed to be 0.50. A type 1 error and a statistical power were set at 0.05 and 0.80, respectively. The critical sample size was computed to be 26. We recruited 55 young and older adults (23 young males and 32 older males). We did not recruit females because of menstrual cycle. Menstrual cycle affects body fluid condition, and impedance measurement is sensitive to it. Therefore, inclusion of data obtained from especially young females has a potential risk of phase angle measurement error. Table 1 shows physical characteristics of the participants. The participants were asked to refrain from strenuous exercise for 24 h, and bathing, eating and drinking for 1 h prior to the experiment. No participants reported any muscle soreness, muscle fatigue, orthopedic or neurological disorders at the time of the experiment. All participants were informed of the purpose and risks of the experiment. A written informed consent to participate in the research was obtained from all participants. The ethics committee of the Shibaura Institute of Technology approved the experimental procedure. This study was performed in accordance with the Declaration of Helsinki.

**TABLE 1:** Anthropometric data, phase angle, twitch contractile properties, muscle strength, and neuromuscular activity of the participants.

	Mean	SD
Anthropometric data		
Age (yr)	52	26
Height (cm)	168.6	6.6
Weight (kg)	65.3	10.0
BMI (kg/m^2^)	23.0	3.3
Phase angle		
Thigh (degree)	7.0	1.4
Whole-body (degree)	6.0	0.9
Twitch contractile properties		
PT_twitch_ (Nm)	25.4	8.1
RTD_twitch_ (Nm/s)	270	97
TPT_twitch_ (s)	0.096	0.011
Muscle strength		
PT_MVIC_ (Nm)	142	49
RTD (Nm/s)	431	141
Neuromuscular activity		
EMG-RMS_MVIC_ (mV)	0.140	0.059
EMG-RMS_RTD_ (mV)	0.095	0.048
nEMG-RMS_RTD_ (%MVIC)	66.8	18.5

SD, standard deviation; PT, peak torque; RTD, rate of torque development; TPT, time-to-peak torque; MVIC, maximal voluntary isometric contraction; EMG-RMS, root mean square value of electromyographic activity; nEMG-RMS_RTD_, EMG-RMS_RTD_ normalized by EMG-RMS_MVIC_.

### Experimental procedures

Experimental procedure and settings conform to our previous study (Hirata et al., 2022). Briefly, after the explanation of the experiment, the participants lay supine on the stretching mat for 10 min. The whole-body and thigh PhA measurements by BIA were performed in supine position three times for each PhA. The participants sat on a dynamometer seat (CON-TREX MJ; Physiomed, Germany) to assess twitch contractile properties of the knee extensors twice by electrical stimulation. Then, voluntary muscle strength and neuromuscular activity of the knee extensors during maximal voluntary isometric contraction (MVIC) were measured. For maximal muscle strength measurement, the participants performed 4-s MVIC twice with at least 1-min rest between the contractions. For explosive muscle strength measurement, the participants performed 1-s brief MVIC 10 times with 20-s rest periods between the contractions.

### Phase angle measurement

Details of the measurement are described elsewhere (Yamada et al., 2010). Briefly, electrodes for current injection (20 × 20 mm, Red Dot; 3M, United States) were placed over the dorsal surfaces of the right hand and right foot. Sensing electrodes (20 × 20 mm, Red Dot; 3M, United States) were attached on the dorsal surface of the right wrist and right ankle for whole-body PhA measurement, and attached on the right greater trochanter and the lateral aspect of the knee joint space of the right lower limb for thigh PhA measurement. By using SFB7 (ImpediMed, Australia), reactance and resistance of the whole-body and thigh were measured three times. PhA was calculated as [arctangent (reactance/resistance) × 180°/π] for each measurement at single frequency of 50 kHz. Mean values of three values for whole-body and thigh PhA were used for further analyses.

### Twitch contractile properties measurement

The participants sat on the dynamometer seat with 80° hip flexion and 90° knee flexion. Their trunk, pelvis and ankle were fixed to the dynamometer seat and lever arm with seat belts and non-elastic straps. The rotational axes of the dynamometer lever arm and knee joint were visually aligned. This sitting posture was same throughout the following measurements: twitch contractile properties, maximal muscle strength, and explosive muscle strength. Stimulation electrodes were attached over the femoral triangle for cathode (20 × 20 mm, Red Dot; 3M, United States) and the lateral aspect of the hip between the greater trochanter and the iliac crest for anode (40 × 50 mm, Natus^®^ Disposable Adhesive Electrodes; Natus Manufacturing Limited, Ireland), in order to stimulate the femoral nerve. Resting twitch response of the knee extensors was elicited by singlet electrical stimulation of 200-μs duration using a constant-current variable voltage stimulator (DS7AH; Digitimer Ltd., United Kingdom). The voltage was set at 400 V. Stimulation intensity was set at 1.2 times the electrical current determined by increasing stimulation current by 10 mA until a plateau in the twitch response was observed. Stimulation intensity was 241 ± 91 mA (mean ± standard deviation). The participants were asked to fully relax during the electrical stimulation measurement. Also, no baseline fluctuation of the torque signal was assured. Twitch peak torque (PT_twitch_) was determined as the difference between maximal knee extension twitch torque and baseline torque. Time-to-peak twitch torque (TPT_twitch_) was computed as the time interval from the onset of twitch torque to the time point at which maximal twitch torque was observed. Rate of twitch torque development (RTD_twitch_) was calculated by dividing PT_twitch_ by TPT_twitch_. Mean values of two twitch responses were used for further analyses. Using LabChart software (ver.8; ADInstruments, Australia), the torque signal was stored on a personal computer through an A/D converter (PowerLab 16/35; ADInstruments). The sampling frequency was at 2 kHz, and was filtered with 500 Hz lowpass filter.

### Electromyography settings

In order to evaluate neuromuscular activity of the knee extensors, a surface EMG system (Bagnoli 8 EMG System; Delsys Inc., Boston, MA, United States) with pre-amplified bipolar active surface EMG electrodes (electrode shape, parallel bar; electrode size, 1 × 10 mm; interelectrode distance, 10 mm; DE-2.1, Delsys Inc.) was used. After shaving, abrasion, and cleaning with alcohol, the electrodes were placed over the rectus femoris (RF), vastus lateralis (VL), and vastus medialis (VM). The longitudinal locations of the electrodes were at proximal 50% of the thigh length (distance between the greater trochanter and the lateral aspect of the knee joint space) for RF, 30% for VL, and 80% for VM. The transverse location of each electrode was at the center of muscle width. Using an ultrasonographic device (ACUSON S2000; Siemens Medical Solutions, Ann Arbor, MI, United States), direction of the electrode was aligned with the fascicle direction of each muscle. The reference electrode was attached on the left medial malleolus. The EMG signal was acquired at 2 kHz, and was filtered with 20–450 Hz bandpass filters.

### Maximal muscle strength measurement

Prior to the actual measurement, submaximal and maximal muscle contractions were performed as warm-up. Submaximal contraction intensity was approximately 20, 50, and 80% MVIC, and the participants performed once at each intensity. After that, several muscle contractions with maximal effort were conducted to familiarize to the actual measurement. The participants performed 4-s MVIC twice with at least 1-min rest between the trials. Strong verbal encouragement and visual feedback of the torque signal were given for the participants. The peak torque of MVIC (PT_MVIC_), the difference between the base line torque and the highest torque value during knee extension with maximal effort, was calculated as maximal muscle strength. Since difference between two values of PT_MVIC_ was less than 10% of the higher one, we judged MVIC measurement was reliable in accordance with a previous study (Akagi et al., 2020). Maximum value of PT_MVIC_ among two trials was used for further analyses. Root mean square value of EMG signal (EMG-RMS) during MVIC for maximal muscle strength measurement was assessed over 500-ms time window. The end point of the time-window was set at time point at which the highest torque value was observed. Mean value of EMG-RMSs for three muscles (RF, VL, and VM) (EMG-RMS_MVIC_) was used for further analyses.

### Explosive muscle strength measurement

The participants exerted 1-s brief MVIC 10 times with 20-s rest between the trials in accordance with previous studies (Maffiuletti et al., 2016; Hirata et al., 2022). They were instructed to exert force as fast and hard as possible. If pre-activation (>3% of EMG-RMS_MVIC_) or countermovement (>0.6 Nm torque variation) in the 200 ms prior to onset of contraction was observed, or peak torque did not reach 70% of PT_MVIC_, the corresponding trials were excluded from analyses. The onset of contraction was defined as the last trough before torque deflection above the range of the baseline noise (0.6 Nm) of the time-torque curve. Rate of torque development (RTD) was analyzed for the three brief MVICs which contain the highest instantaneous RTDs, which were calculated from differential waveform of the time-torque curve. RTD was computed as the average slope of the time-torque curve over the time intervals of 0–200 ms (0 ms: onset of contraction). EMG-RMS values during the brief MVIC for explosive muscle strength measurement were calculated over the same time intervals of RTD (i.e., 0–200 ms) from the onset of EMG activity. EMG onset was defined as the last trough before EMG signal deflection above the range of the baseline noise (3% of EMG-RMS_MVIC_) of the rectified EMG signals. EMG-RMSs obtained from RF, VL, and VM were averaged as EMG-RMS_RTD_. Each mean value of RTD and EMG-RMS_RTD_ values calculated from the selected three trials were used for further analyses. Absolute value of EMG-RMS_RTD_ was normalized by EMG-RMS_MVIC_ (nEMG-RMS_RTD_).

### Habitual physical activity measurement

The participants were asked to wear a device (Active style Pro HJA-750C, Omron Health Care, Japan) to quantify their habitual physical activity for 11 days except when bathing or sleeping. Habitual physical activity measurement was conducted after the above-mentioned measurements (i.e., muscle strength measurement, etc.). The data from days at which the participants wore the device for >500 min/day as recorded in a diary were used for the analyses. Analyzable data period ranged from 4 to 11 days. Based on metabolic equivalents (METs) measured by the activity monitor in 0.1 METs increments, physical activity was determined per day at three levels: light (1.1–2.9 METs), moderate (3.0–5.9 METs), and vigorous intensities (≥6.0 METs) (Gando et al., 2010).

### Statistical analyses

Pearson product-moment correlation analysis was performed to test the association of PhA with the measurement variables. A stepwise multiple linear regression analysis using the measures of voluntary muscle strength (PT_MVIC_ and RTD) as the independent variables was conducted. The dependent variables for the multiple regression analyses were the acquired data (twitch contractile properties, EMG activities, and anthropometric data) which significantly correlated with PT_MVIC_ or RTD by the Pearson product-moment correlation analysis. Whole-body and thigh PhA were entered into the multiple regression analyses irrespective of the statistical results of the Pearson product-moment correlation analysis with PT_MVIC_ or RTD, because the goals of the present study were to investigate whether whole-body or thigh PhA is the best predictor of muscle strength of the knee extensors. Additionally, age was also entered into the multiple regression analyses [as a dummy variable (young = 0, older = 1)], since age may significantly influence the muscle strength of the knee extensors in case of presence of young and older participants. The relative contribution of each selected independent variable to estimation of PT_MVIC_ or RTD in the multiple regression equation was calculated as the product of the standardized *β* in the multiple regression equation and the simple regression coefficient in the relationship with PT_MVIC_ or RTD for each selected independent variable, expressed as percentage (Miyatani et al., 2004; Akagi et al., 2018). For aid to better interpretation of the present data, age-related difference in the measurement variables was tested by an independent *t*-test as supplementary data. Statistical significance was set at *p* < 0.05, and effect sizes (*r* for Pearson product-moment correlation analysis; *d* for *t*-test) were reported. All statistical analyses were performed using statistical software (SPSS Statistics 28.0, IBM Japan, Japan).

## Results

### Correlations of phase angle with twitch contractile properties and neuromuscular activity

**FIGURE 1:**
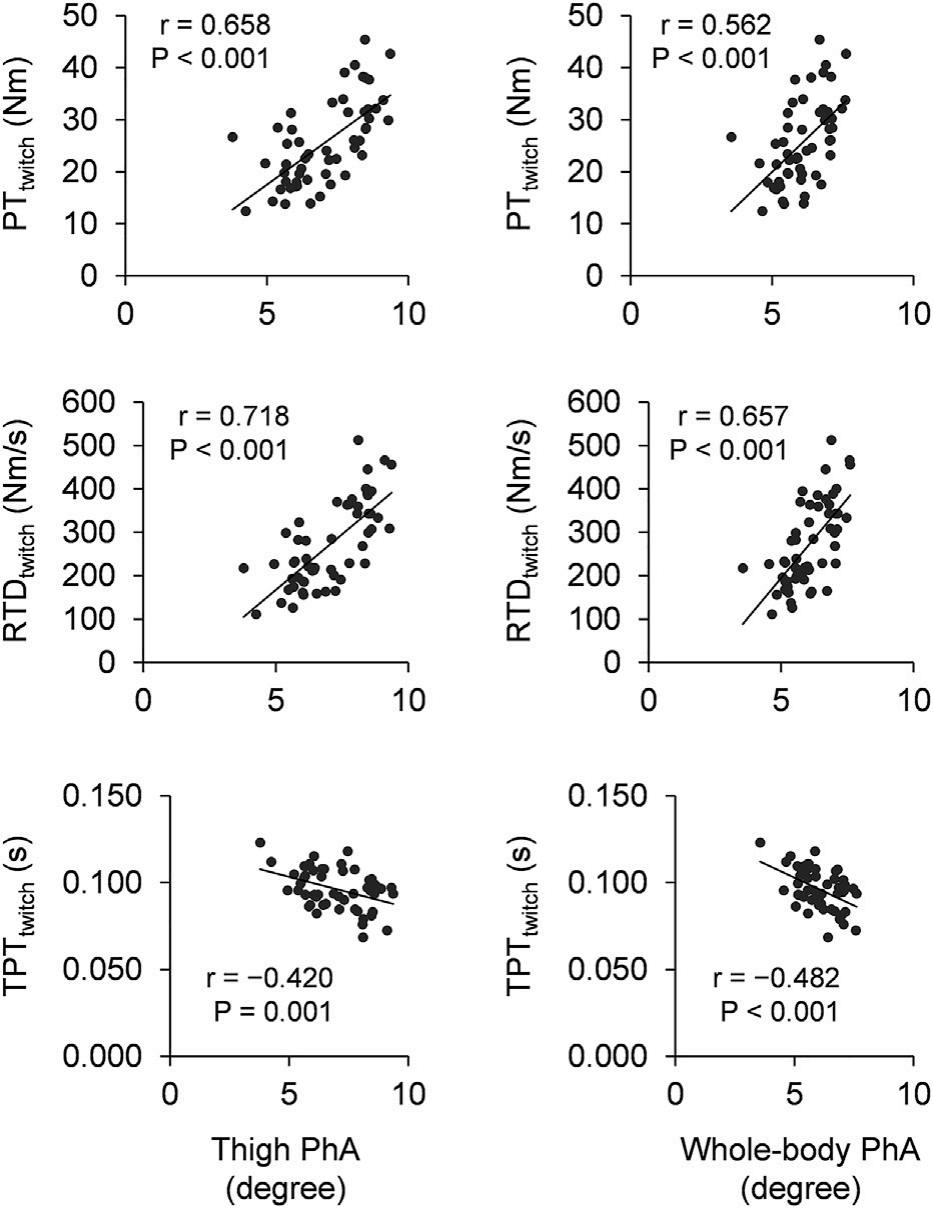
Scatter plots of thigh phase angle (left column) and whole-body phase angle (right column) with twitch contractile properties. PT: peak torque, RTD: rate of torque development, TPT: time-to-peak torque, PhA: phase angle.

Figure 1 shows the scatter plots between PhA and twitch contractile properties. Thigh PhA significantly correlated positively with PT_twitch_ (*r* = 0.658 and *p* < 0.001) and RTD_twitch_ (*r* = 0.718 and *p* < 0.001), negatively with TPT_twitch_ (*r* = −0.420 and *p* = 0.001). Similarly, whole-body PhA significantly correlated positively with PT_twitch_ (*r* = 0.562 and *p* < 0.001) and RTD_twitch_ (*r* = 0.657 and *p* < 0.001), negatively with TPT_twitch_ (*r* = −0.482 and *p* < 0.001). For neuromuscular activity, thigh PhA did not significantly correlate with EMG-RMS_MVIC_ (*r* = 0.214 and *p* = 0.117), EMG-RMS_RTD_ (*r* = 0.113 and *p* = 0.412), or nEMG-RMS_RTD_ (*r* = −0.025 and *p* = 0.856). Similarly, whole-body PhA did not significantly correlate with EMG-RMS_MVIC_ (*r* = 0.188 and *p* = 0.170), EMG-RMS_RTD_ (*r* = 0.072 and *p* = 0.599), or nEMG-RMS_RTD_ (*r* = −0.035 and *p* = 0.802).

### Correlations of phase angle with maximal and explosive muscle strength

**FIGURE 2:**
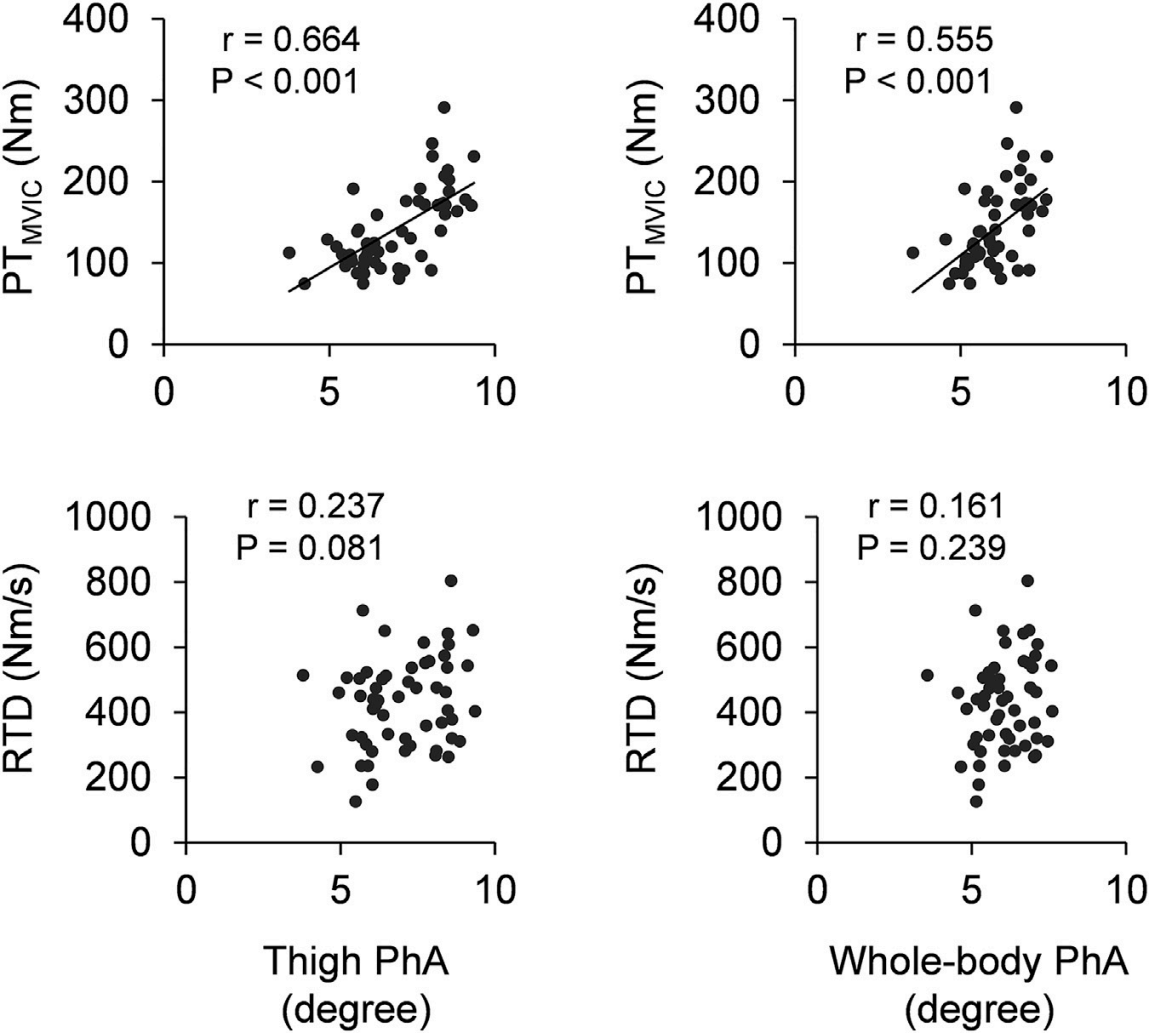
Scatter plots of thigh phase angle (left column) and whole-body phase angle (right column) with maximal muscle strength (PT_MVIC_) and explosive muscle strength (RTD). PT: peak torque, MVIC: maximal voluntary isometric contraction, RTD: rate of torque development, PhA: phase angle.

Figure 2 shows the scatter plots of PhA with maximal muscle strength and explosive muscle strength. Significant positive correlations of thigh and whole-body PhA with PT_MVIC_ were observed (*r* = 0.664 and *p* < 0.001 for thigh PhA; *r* = 0.555 and *p* < 0.001 for whole-body PhA). Thigh and whole-body PhA did not significantly correlate with RTD (*r* = 0.237 and *p* = 0.081 for thigh PhA; *r* = 0.161 and *p* = 0.239 for whole-body PhA).

### Multivariate analysis of linear regression model for maximal and explosive muscle strength

Tables 2, 3 show the results of the multivariate analysis of stepwise linear regression model for PT_MVIC_ and RTD, respectively. Candidate variables for the multi regression analysis for PT_MVIC_ were as follows: thigh PhA, whole-body PhA, age, weight, PT_twitch_, and RTD_twitch_. The best fitted model (i.e., model 3; Table 2) accounts for 71.4% variance of PT_MVIC_, and relative contributions of the selected variables were as follows: 49.6% for PT_twitch_, 17.0% for thigh PhA, and 6.4% for Weight. For the multi regression analysis for RTD, candidate variables were as follows: thigh PhA, whole-body PhA, age, weight, PT_twitch_, RTD_twitch_, EMG-RMS_RTD_, and nEMG-RMS_RTD_. The best fitted model (i.e., model 4; Table 3) accounts for 61.7% variance of RTD, and relative contributions of the selected variables were as follows: 24.1% for nEMG-RMS_RTD_, 12.1% for PT_twitch_, 14.9% for Weight, and 13.4% for EMG-RMS_RTD_. As shown above, thigh PhA was selected as a significant variable to predict PT_MVIC_ independent of other variables, but not RTD. Whole-body PhA was not selected as a significant variable to predict PT_MVIC_ or RTD.

**TABLE 2:** Multivariate analysis of linear regression model for maximal muscle strength.

Dependent variable: PT_MVIC_ (Nm) Model		Unstandardized		Standardized		*p*-value
		*B*	SE	*β*	*t*	
1	(Constant)	17.38	12.67		1.37	0.176
*R* ^2^ = 0.661	PT_twitch_	4.91	0.48	0.82	10.32	**<0.001**
2	(Constant)	−15.84	19.48		−0.81	0.420
*R* ^2^ = 0.684	PT_twitch_	4.03	0.61	0.67	6.60	**<0.001**
	Thigh PhA	7.93	3.62	0.22	2.19	**0.033**
3	(Constant)	−75.33	29.77		−2.53	**0.015**
*R* ^2^ = 0.714	PT_twitch_	3.64	0.60	0.61	6.08	**<0.001**
	Thigh PhA	9.11	3.47	0.26	2.63	**0.011**
	Weight	0.93	0.37	0.19	2.55	**0.014**

PT, peak torque; MVIC, maximal voluntary isometric contraction; SE, standard error; PhA, phase angle; RTD, rate of torque development. Candidate variables: age, weight, thigh PhA, whole-body PhA, PT_twitch_, RTD_twitch_. *R* ^2^ was adjusted R square. Bold values represent statistical significance.

**TABLE 3:** Multivariate analysis of linear regression model for explosive muscle strength.

Dependent variable: RTD (Nm/s) Model		Unstandardized		Standardized		*p*-value
		*B*	SE	*β*	*t*	
1	(Constant)	135.34	58.98		2.29	**0.026**
*R* ^2^ = 0.326	nEMG-RMS_RTD_	4.43	0.85	0.58	5.21	**<0.001**
2	(Constant)	−66.89	69.31		−0.97	0.339
*R* ^2^ = 0.494	nEMG-RMS_RTD_	4.68	0.74	0.61	6.33	**<0.001**
	PT_twitch_	7.31	1.69	0.42	4.32	**<0.001**
3	(Constant)	−302.44	98.95		−3.06	**0.004**
*R* ^2^ = 0.567	nEMG-RMS_RTD_	4.66	0.68	0.61	6.82	**<0.001**
	PT_twitch_	6.21	1.60	0.36	3.87	**<0.001**
	Weight	4.05	1.30	0.29	3.13	**0.003**
4	(Constant)	−399.69	99.50		−4.02	**<0.001**
*R* ^2^ = 0.617	nEMG-RMS_RTD_	3.15	0.84	0.41	3.73	**<0.001**
	PT_twitch_	5.69	1.52	0.33	3.74	**<0.001**
	Weight	5.89	1.39	0.42	4.24	**<0.001**
	EMG-RMS_RTD_	961.40	347.90	0.33	2.76	**0.008**

RTD, rate of torque development; SE, standard error; EMG-RMS, root mean square value of electromyographic activity; nEMG-RMS_RTD_, EMG-RMS_RTD_ normalized by EMG-RMS_MVIC_; MVIC, maximal voluntary isometric contraction; PT, peak torque. Candidate variables: age, weight, thigh PhA, whole-body PhA, PT_twitch_, RTD_twitch_, EMG-RMS_RTD_, nEMG-RMS_RTD_. *R* ^2^ was adjusted R square. Bold values represent statistical significance.

### Habitual daily activity

Physical activity in the young participants was 607 ± 64 min/day for light, 84 ± 14 min/day for moderate, and 3 ± 6 min/day for vigorous intensity. In the older participants, 452 ± 93 min/day for light, 76 ± 35 min/day for moderate, and 2 ± 3 min/day for vigorous intensity.

### Age-related differences in the measurement variables

Independent *t*-tests revealed that thigh and whole-body PhA of young adults were larger than those of older adults (Supplementary Table S1). For twitch contractile properties, young adults showed greater PT_twitch_ and RTD_twitch_, and shorter TPT_twitch_ compared with older adults. PT_MVIC_ was greater in young adults than in older adults, although there was no significant difference in RTD between young and older adults. No significant differences were observed for EMG activities between young and older adults.

## Discussion

Main findings of the present study were as follows: 1) Larger thigh and whole-body PhA correlated with greater PT_MVIC_, PT_twitch_, and RTD_twitch_, and with shorter TPT_twitch_, but not with EMG-RMSs. 2) Thigh PhA, but not whole-body PhA was selected as the predictor of PT_MVIC_. 3) Thigh or whole-body PhA were not selected as the predictor of RTD. These findings suggest that both thigh and whole-body PhA can associate with twitch contractile properties and maximal muscle strength (PT_MVIC_) of the knee extensors. However, explosive muscle strength (RTD) and neuromuscular activity (EMG-RMS) of the knee extensors would be difficult to predict from thigh or whole-body PhA. Regarding prediction of maximal muscle strength of the knee extensor, thigh PhA is preferable as the predictor rather than whole-body PhA which is used as a widely acknowledged indicator of dynapenia.

To the best of our knowledge, this study is the first one to elucidate the association of thigh PhA with maximal muscle strength of the knee extensors in healthy adults (Figure 2). This is in line with our hypothesis and a previous finding of study of Wada et al. (2020) which reported correlation between PhA and isometric knee extension torque in the patients with knee osteoarthritis. PhA is suggested to relate with muscle mass (Di Vincenzo et al., 2021). Because muscle mass is one of the major determinant factors of maximal muscle strength, the association of thigh PhA with maximal muscle strength of the knee extensors could be due to muscle mass of the knee extensors. The present study was also revealed the association of thigh PhA with twitch contractile properties of the knee extensor (Figure 1). Because PT_twitch_ and RTD_twitch_ are also dependent on muscle mass, the correlations of PhA with PT_twitch_ and RTD_twitch_ are likely reasonable. Thigh PhA did not correlate with neuromuscular activity during MVIC (i.e., EMG-RMS_MVIC_). PhA is suggested to reflect cell membrane integrity and cell function (Norman et al., 2012). Since cellular membrane properties can be one of the influencing factors of neuromuscular activity (Farina et al., 2004), it could be expected that PhA associates with EMG activity. Indeed, a previous study (Yamada et al., 2022) reported that EMG amplitude of the plantar flexors was correlated with membrane capacitance which is bioelectrical impedance spectroscopy index closely related to PhA. On the other hand, surface EMG reflects not only peripheral properties but also central properties of the neuromuscular system (Farina et al., 2004). Hence, even if PhA would associate to cellular membrane function, its contribution to EMG activity may be small due to large influence of the central nervous system for the knee extensors. Collectively, the associations of thigh PhA with maximal muscle strength may be due to factors which relates to twitch contractile properties but not neuromuscular activity, such as muscle mass.

Like the correlation of thigh PhA, whole-body PhA correlated with maximal muscle strength (Figure 1) and twitch contractile properties but not neuromuscular activity. Regarding only the single regression analysis results, maximal muscle strength of the knee extensors is considered to be predictable from whole-body PhA. However, the multivariate analysis of stepwise linear regression model revealed that not whole-body PhA but thigh PhA was selected as the predictive variable for maximal muscle strength of the knee extensors (Table 2). This is reasonable because whole-body PhA is not a measure of thigh segment. Yamada et al. (2021) also suggested that locomotor function evaluated by timed-up-and-go test was more highly correlated with PhA measured from lower limb compared with PhA measured from whole-body. From the present and previous findings, a variable to predict the lower limb function would be more preferable to PhA measured from the corresponding segment (i.e., thigh and lower limb) than whole-body. Although whole-body PhA is used as a widely acknowledged indicator of sarcopenia (Cruz-Jentoft et al., 2019), considering a large decrement in thigh muscularity with age (Frontera et al., 2000; Nogueira et al., 2013) and an importance of thigh muscularity for independence of older adults (Hasegawa et al., 2008), PhA obtained from lower limb would be recommended for assessment of functionality of older adults.

RTD was not correlated with thigh or whole-body PhA (Figure 2). No correlation between PhA and RTD may be because neuromuscular activity largely contributes to explosive muscle strength production (Maffiuletti et al., 2016). Indeed, the multivariate analysis of stepwise linear regression model revealed that nEMG-RMS_RTD_ is the strong indicator of RTD (Table 3). Especially when exerting explosive muscle strength, central nervous system (e.g., motor unit discharge frequency) plays an important role (Klass et al., 2008; Morel et al., 2015). As mentioned earlier, there may be a possibility to connect PhA with peripheral properties of neuromuscular system but not central properties of that. Therefore, PhA could not correlate with EMG activity during RTD leading to no association between PhA and RTD. On the other hand, potential positive association between thigh PhA and RTD (Figure 2) and significant negative associations of thigh and whole-body PhA with TPT_twitch_ (Figure 1) were observed in the present study. TPT_twitch_ becomes long with age (Hirata et al., 2022) because TPT_twitch_ is shorter in a muscle containing greater type II fiber (Harridge et al., 1996) and type II fiber content is decreased with age (Lexell, 1995). Also, PhA becomes smaller with age possibly because PhA is directly related to amount and functional status of cell membranes and these decrease with age (Barbosa-Silva et al., 2005). These age-related differences in TPT_twitch_ and PhA were also shown in the present study (Supplementary Table S1). Hence, the association of larger PhA with shorter TPT_twitch_ observed among young and older adults may be due to a common factor of age-related change in TPT_twitch_ and PhA. Collectively, although PhA might associate with rapid force production ability of intrinsic muscle contractile properties, thigh or whole-body PhA cannot predict voluntary explosive strength of the knee extensors owing to neuromuscular activity, which strongly associates with voluntary explosive muscle strength but not PhA.

The present findings suggest the advantage of measurement for PhA to estimate maximal voluntary muscle strength. Although healthy individuals can perform vigorous muscle contraction to quantify maximal muscle strength, necessity for the quantification is not only for healthy individuals but also older adults and patients with orthopedic or cognitive disorders. However, it is predictable that such people could not exert muscle force with maximal effort or even impossible to appropriate muscle contraction. Because PhA can be assessed non-invasively and quickly by BIA without muscle contraction, assessment of PhA may be a valuable way to evaluate muscle force generating capacity for such people.

The present study has several limitations. First, thigh PhA is a segment-level measurement but not a specific muscle group-level such as the knee extensors. Hence, non-target muscle groups (e.g., the hamstring muscles) can influence the association of thigh PhA with the measurement variables. Nevertheless, significant correlations of PhA and maximal muscle strength and twitch contractile properties of the knee extensors were confirmed. Hence, we believed that thigh PhA could sufficiently reflect the characteristics of the knee extensors. Second, we investigated the associations of PhA only among the pooled group but not separate age group. This is because the main goal of this study was to investigate association of phase angle with neuromuscular properties in healthy adults regardless of age. On the other hand, as significant age-related differences in the measurement variables were shown (Supplementary Table S1), the significant correlations of PhA with the measurement variables observed in this study may be merely due to acting age as the cofounding factor. However, the multivariate analysis of stepwise linear regression model suggested that age was not selected as the predictive factor. This indicates that age is considered not to be the major factor of the association of PhA with the knee extensor contractile properties, at least the voluntary muscle strength. Therefore, it can be said that the significant correlations were observed due to individual differences rather than age-related differences. Third, skin impedance verification was not conducted in the present study. This may influence EMG measurement reliability. However, the present study was used a pre-amplified active EMG electrode which is less susceptible to noise. Additionally, we visually assured that signal-to-noise ratio was not problematic. Therefore, we believed that EMG signal was of sufficient quality to analyze. Lastly, this study recruited only males but not females. Because sex-related difference in change of PhA with aging (Saragat et al., 2014) and that in muscle strength and physical performance (Lu et al., 2020) were suggested, there might also be sex-related differences in the association of PhA with neuromuscular properties. Hence, it may be necessary to explore whether the influence of PhA on the knee extensor muscle strength is sex-dependent.

## Conclusion

The purpose of the present study was to investigate associations of PhA with neuromuscular properties of the knee extensors. Thigh and whole-body PhA correlated with PT_MVIC_ and twitch contractile properties (PT_twitch_, RTD_twitch_, and TPT_twitch_) but not RTD or EMG activity (EMG-RMS_MVIC_, EMG-RMS_RTD_, and nEMG-RMS_RTD_) of the knee extensors. The multiple regression analysis revealed that thigh PhA but not whole-body PhA was selected as a significant variable to predict PT_MVIC_ independent of other variables. These results suggest that both thigh and whole-body PhA can associate with contractile properties but not neuromuscular activity of the knee extensors. Regarding prediction of maximal knee extensor strength, thigh PhA is preferable as the predictor rather than whole-body PhA which is used as a widely acknowledged indicator of sarcopenia.

## Data Availability

The raw data supporting the conclusion of this article will be made available by the authors, without undue reservation.
