# Supplementary material for: Can phase angle from bioelectrical impedance analysis associate with neuromuscular properties of the knee extensors?
Source: Front Physiol. 2022 Aug 11;13:965827. doi: 10.3389/fphys.2022.965827 (PMC9403265; doi:10.3389/fphys.2022.965827)
Supplement: Supplementary file 1 [file Table1.DOCX]

**Supplementary Table 1**. Anthropometric data, phase angle, twitch contractile properties, muscle strength, and neuromuscular activity of the young and older participants

|  |  | Young | | Older | | Independent t-test | |
| --- | --- | --- | --- | --- | --- | --- | --- |
|  |  | Mean | SD | Mean | SD | *P*-value | Cohen’s *d* |
| Anthropometric data | |  |  |  |  |  |  |
|  | Age (yr) | 21 | 2 | 74 | 6 | **<0.001** | −11.64 |
|  | Height (cm) | 172.1 | 5 | 166.1 | 6.6 | **0.001** | 1.00 |
|  | Weight (kg) | 64.8 | 10.4 | 65.6 | 9.8 | 0.750 | −0.09 |
|  | BMI (kg/m^2^) | 21.9 | 3.7 | 23.7 | 2.8 | **0.043** | −0.57 |
| Phase angle | |  |  |  |  |  |  |
|  | Thigh (degree) | 8.3 | 0.6 | 6.1 | 0.9 | **<0.001** | 2.89 |
|  | Whole-body (degree) | 6.8 | 0.5 | 5.5 | 0.6 | **<0.001** | 2.14 |
| Twitch contractile properties | |  |  |  |  |  |  |
|  | PT_twitch_ (Nm) | 32.5 | 6.4 | 20.4 | 4.6 | **<0.001** | 2.21 |
|  | RTD_twitch_ (Nm/s) | 362 | 67 | 204 | 48 | **<0.001** | 2.81 |
|  | TPT_twitch_ (s) | 0.090 | 0.010 | 0.101 | 0.010 | **<0.001** | −1.06 |
| Muscle strength | |  |  |  |  |  |  |
|  | PT_MVIC_ (Nm) | 181 | 47 | 114 | 24 | **<0.001** | 1.90 |
|  | RTD (Nm/s) | 464 | 146 | 408 | 135 | 0.150 | 0.40 |
| Neuromuscular activity | |  |  |  |  |  |  |
|  | EMG-RMS_MVIC_ (mV) | 0.145 | 0.062 | 0.136 | 0.057 | 0.603 | 0.14 |
|  | EMG-RMS_RTD_ (mV) | 0.095 | 0.05 | 0.094 | 0.048 | 0.925 | 0.03 |
|  | nEMG-RMS_RTD_ (%MVIC) | 66.3 | 17.1 | 67.2 | 19.8 | 0.863 | −0.05 |

SD: standard deviation, PT: peak torque, RTD: rate of torque development, TPT: time-to-peak torque, MVIC: maximal voluntary isometric contraction, EMG-RMS: root mean square value of electromyographic activity, nEMG-RMS_RTD_: EMG-RMS_RTD_ normalized by EMG-RMS_MVIC_
